# Quantification of Dissolved and Particulate Polyunsaturated Aldehydes in the Adriatic Sea

**DOI:** 10.3390/md9040500

**Published:** 2011-03-29

**Authors:** Charles Vidoudez, Raffaella Casotti, Mauro Bastianini, Georg Pohnert

**Affiliations:** 1 Institute of Inorganic and Analytical Chemistry, Friedrich Schiller University, Lessingstr. 8, D-07743 Jena, Germany; E-Mail: cvidoudez@oeb.harvard.edu (C.V.); 2 Stazione Zoologica A. Dohrn, Villa Comunale I80121 Napoli, Italy; E-Mail: raffa@szn.it (R.C.); 3 Istituto di Scienze Marine CNR, Castello 1364/a I30122 Venice, Italy; E-Mail: mauro.bastianini@ismar.cnr.it (M.B.)

**Keywords:** chemical defense, aldehydes, plankton interactions, oxylipins, heptadienal

## Abstract

Polyunsaturated aldehydes (PUA) are supposed to play critical roles in chemically-mediated plankton interactions. Laboratory studies suggest that they act as mediators of chemical defense and chemical communication. PUA are oxylipins containing an α,β,γ,δ–unsaturated aldehyde structure element and are mainly found in diatoms. We present here a detailed surface mapping of PUA during a spring bloom of the diatom *Skeletonema marinoi* in the Adriatic Sea. We monitored dissolved PUA, as well as particulate PUA, which are produced by phytoplankton after cell disintegration. Our survey revealed a patchy distribution of PUA and shows that at most stations *S. marinoi* is the major contributor to the overall PUA. Our data also suggest that lysis of a diatom bloom can contribute significantly to the dissolved PUA concentrations and that other producers, which are smaller in cell size compared to diatoms, have to be taken into account as well if the total PUA content of marine samples is considered. The analyses of samples collected in deeper water suggests that diatom contribution to PUA decreases with depth, while smaller-sized unidentified organisms take place as dominant contributors to the PUA concentrations.

## 1. Introduction

Polyunsaturated aldehydes (PUA) from diatoms are fatty-acid-derived secondary metabolites with an α,β,γ,δ-unsaturated aldehyde structure element. A survey of cultured diatom strains reported that roughly a third of marine diatoms are capable of producing PUA [1], and if this holds true for natural conditions, it can be assumed that these compounds are quite common in the ocean, mainly in coastal waters, where diatoms form intense blooms.

Several roles in ecological interactions have been attributed to diatom-produced PUA [2]. Initially, 2,4-decadienal and 2,4,7-decatrienal were identified as active principles that impair the hatching success of copepods feeding on a diatom-dominated bloom in the Adriatic Sea [3]. Subsequently, several structurally-related metabolites were identified from marine diatoms that all exhibited inhibitory activity on the hatching success of copepod and sea urchin eggs [4]. In addition, PUA in maternal diets of copepods induce malformations in the copepod offspring [5]. Studies have now expanded to demonstrate inhibitory effects of PUA on the growth of cultured phytoplankton [6,7] and bacteria [8,9]. At relatively high concentrations, PUA also cause a genotoxic effect by interfering with the cell cycle progression or by inducing DNA degradation and chromatin fragmentation; a mechanism closely resembling apoptosis of higher organisms [6].

Apart from a direct toxic effect, alternative roles of PUA have been explored in several culture studies. 2,4-Decadienal, for instance, appears to be involved in a stress-signaling mechanism in the diatom *Phaeodactylum tricornutum*, triggering cytosolic Ca^2+^ mobilization and NO production [10]. At relatively high concentrations, this leads to the propagation of the cell death signal and massive decay of the diatom population. In accordance with the idea of an induced cell death, Vidoudez and Pohnert reported that nanomolar concentrations of PUA in the medium can accelerate cell death of a diatom culture in the late stationary growth phase [11]. Sublethal concentrations of this PUA also induce resistance to further doses, conferring an advantage to diatoms over competing species [10].

In diatoms, the production of PUA is triggered by cell disruption and is dependent on a complex enzyme cascade [12]. Within seconds after mechanical disruption of diatom cells lipases release polyunsaturated fatty acids from phospho- and galactolipids [13,14]. These free fatty acids are then transformed by lipoxygenase/lyase enzyme activities into PUA [15,16]. These processes lead to the release of high local PUA concentrations around damaged diatom cells. Until recently, it was believed that PUA are only produced after cell disruption, as it occurs during mastication by grazers and/or cell lysis. However, late-stationary cultures of the diatom *Skeletonema marinoi* also release PUA into the medium without concomitant grazing or cell lysis [11]. This supports their role as intra-population signaling molecules.

Most of the conclusions about the role of PUA are drawn from laboratory experiments where often comparably high doses of these metabolites were added to test organisms. Only a few studies report the PUA production of natural plankton assemblages after cell disruption [17–19]. No field data are available on the actual PUA concentrations in the water, which are relevant if bloom termination or other cell-cell communication strategies are concerned. Here we present data from an extended field survey of PUA in the northern Adriatic Sea (Mediterranean) during a bloom of the diatom *S. marinoi*. We monitored the PUA which were produced after mechanical disruption of the cells (hereafter particulate PUA) as well as PUA that were present in the water (dissolved PUA).

## 2. Results and Discussion

### 2.1. Particulate PUA

The modified protocols for the analysis of dissolved and particulate PUA allow the qualitative and quantitative survey of these metabolites in field samples. It was possible to monitor particulate PUA after filtration of less than one liter of plankton samples thereby requiring a minimal sampling effort. The filtration and derivatization steps are easy to perform and therefore suitable for sample preparation on board. Since the derivatized samples are stable they can be stored and transferred to an analytical lab without loss of accuracy. The short time needed for sample preparation allows a sampling frequency of less than 1 h.

**Figure 1.**
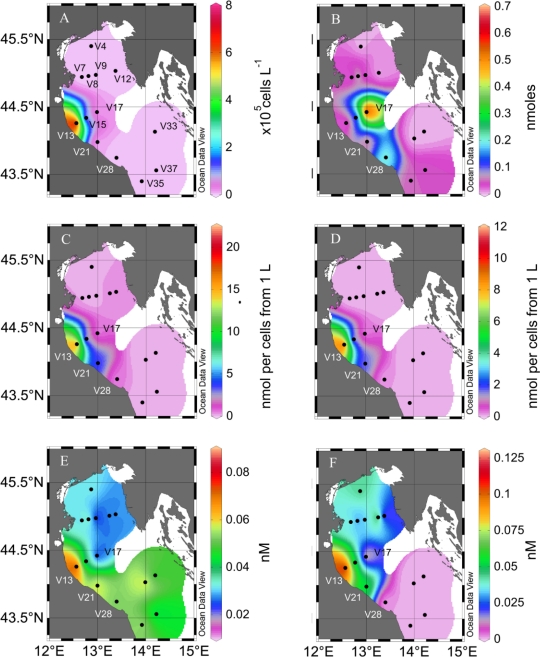
Surface distribution (0–1 m) of dissolved and particulate PUA in the Adriatic Sea in February 2008. (**A**), *S. marinoi* cell density. (**B**), particulate heptadienal produced by the 1.2 μm > x > 0.2 μm fraction from 1 L of seawater. (**C**), particulate heptadienal and (**D**), octadienal produced by the >1.2 μm fraction obtained from 1 L of seawater. (**E**), dissolved heptadienal and (**F**), octadienal concentrations in the seawater. The closed circles indicate the sampling stations. Note the different scaling. The colored surfaces are representation of the data presented in Table 1 and are only used to facilitate the visualization. They are not extrapolations of the concentrations.

In our survey of the northern Adriatic Sea, the highest density of *Skeletonema marinoi* was found at station V13 (0.7 10^6^ cell L^−1^) (Figure 1A, Table 1), which is often the site of a recurrent winter bloom of this diatom (M. Bastianini, pers. comm.). *S. marinoi* dominated phytoplankton, accounting for 30% of total phytoplankton counts and >95% of all diatom species at this station. The cells in the >1.2 μm fraction (as defined by the cutoff of the filters used) showed a very high particulate PUA production. If normalized by the filtered volume of seawater, the potential production by the plankton in 1 L was 18 nmol heptadienal and 10 nmol octadienal (referred as nmol per cells from 1 L, Figure 1 C and D, Table 1).

If it is assumed that *S. marinoi* is the major PUA producer in the samples, the particulate PUA production normalized to cells from this alga would be 22 fmol *S. marinoi* cell^−1^ and 13 fmol octadienal *S. marinoi* cell^−1^. These values are well in accordance with results from laboratory experiments where *S. marinoi* produced heptadienal and octadienal as major PUA reaching concentrations in the range of below 0.1 to above 25 fmol cell^−1^ [20,21].

A significant direct correlation between particulate PUA and *S. marinoi* concentrations was observed (Pearson’s p value <0.0001 for both heptadienal and octadienal, coefficients 0.903 and 0.911, respectively), suggesting that this species was the dominant producer of PUA. However, *S. marinoi* was apparently not the only contributor to particulate PUA production in the Adriatic Sea. In fact, at station V21 3.21 nmol heptadienal and 1.6 nmol octadienal were detected from the cells in 1 L of seawater, while *S. marinoi* was not present (Table 1). PUA production has been reported from other non-diatom phytoplankton, such as *Phaeocystis pouchetii* which is a source for decadienal [22] and it is thus likely that additional producers can occur locally at specific field sites. The sample at station V21 contained predominantly *Emiliania huxleyi* which, however, has never been reported to produce PUA, suggesting that some other yet unidentified species are responsible for the detected PUA.

**Figure 2.**
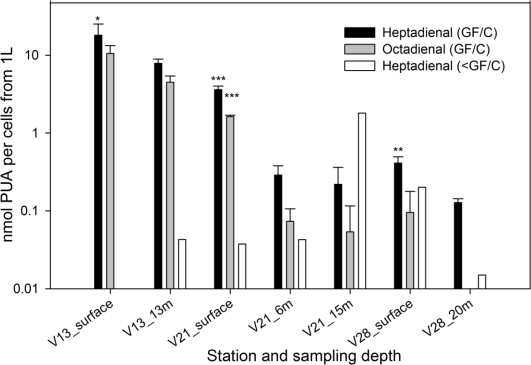
Production of PUA by cells from 1 L of seawater collected at different depths from different size fractions. All values are average ± standard deviation (n = 3), except for the heptadienal < GF/C (white bars), where n = 1. Statistical evaluation was only performed for heptadienal GF/C and octadienal GF/C. The values that are significantly different from the other depth at the same station are indicated by stars (t-test was used for the comparison of data from two different depth at V13 as well as V28, one-way ANOVA followed by Holm-Sidak comparison was used for measurements at three different depths at station V21 * p < 0.05, ** p < 0.01, *** p < 0.005). Note the logarithmic scale.

At three stations, also intermediate depths were sampled. In all cases, particulate PUA production from cells >1.2 μm was significantly lower in deeper water when compared to the surface (Figure 2), coincident with the lower diatom contribution at depth (Table 1). This may suggest that diatoms are replaced by other organisms producing lower amounts or no PUA at depth. Since many factors are known to modulate PUA production, including nutrient availability, cell growth stage and strain variability another explanation for the observed trend might be that diatoms at depth were producing lower amounts of PUA per cell when compared to the surface [20,21].

At station V17, high amounts of particulate heptadienal (0.5 nmol cells^−1^ from 1 L, Figure 1B) were produced from cells in the 1.2 μm > x > 0.2 μm size fraction despite no *S. marinoi* cells were detected. In addition, no octadienal was found in these samples, which further supports that other species were responsible for the PUA production: In fact, *S. marinoi* always produces a mixture of heptadienal and octadienal and never one PUA alone [11,21]. Diatoms have been considered as the major source for PUA in phytoplankton, but our findings now demonstrate that also other cells that are well below the typical size of diatoms may at times represent additional sources of PUA and have to be considered. These additional PUA producers are below the size which is usually grazed by copepods, yet in the light of the multiple observed effects of this compound class, PUA from these sources might still influence the plankton community structure. Interestingly, heptadienal concentrations in the size fraction between 0.2 and 1.2 microns increased with depth at two stations (Figure 2), suggesting that the alternative species responsible for heptadienal production may be more adapted to deeper conditions than diatoms.

From our data it is evident that particulate PUA are occurring with explicit local concentration maxima and that patchy chemical landscapes exist in the Adriatic Sea (Figure 1). This has major consequences on the chemical ecology of plankton, since local PUA concentrations can be much higher than the ones averaged over a large volume of seawater. Obviously, chemically mediated interactions have to be investigated using a defined sampling regime and preferably ecological and chemical investigations have to be performed using the same samples. Thus, for example, field experiments to elucidate the potential impact of PUA against copepods need to consider particulate PUA production in all algae that serve as food sources of the copepods. Diatom cell counts alone will not be helpful to predict PUA concentrations since additional PUA producers obviously play important roles.

### 2.2. Dissolved PUA

We report for the first time the occurrence of dissolved PUA in natural seawater samples. The detection limit of our method is 10 pM for dissolved PUA which makes the analytical survey possible with a relatively small sample volume (few liters at the most). We observed a direct correlation between *S. marinoi* cell numbers and concentrations of dissolved octadienal (Pearson’s correlation coefficient 0.596, p < 0.01)(Figure 1F). Decreasing dissolved octadienal PUA concentrations were observed when sampling farther away from the *S. marinoi* bloom. In contrast, the additional heptadienal production from organisms different than diatoms might be responsible for the observed lack of correlation of this PUA with *S. marinoi* cell numbers (Pearson product moment coeff. 0.298, p value ≥ 0.1) (Figure 1E, Table 1).

Dissolved PUA never reached concentrations comparable to those of PUA liberated from wounded phytoplankton cells (Table 1). Dissolved heptadienal and octadienal concentrations were *ca*. 0.5% and 1% of the particulate PUA concentrations at stations where *S. marinoi* dominated. This may suggest that not the whole enzymatic potential for PUA production was contributing to PUA release or else that only a fraction of the diatom cells was releasing PUA. Previous studies from cultures show that PUA may be released without cell disruption by grazing or cell death [11]. The PUA concentrations observed in our field survey are lower compared to the nearly 100 nM that were reached in *S. marinoi* batch cultures, which might be due to significant dilution effects in the moving water bodies [11]. It should be noted that the measured concentrations only reflect PUA at a given time, without considering the production and turnover rates of these reactive compounds, which are degraded abiotically and biotically in the water. As a consequence, the actual release rates might be significantly underestimated if only the dissolved PUA concentrations are considered. Surprisingly, high amounts of dissolved PUA were also observed at stations were no or little *S. marinoi* was present. In fact, at several stations dissolved heptadienal and octadienal were found even if no particulate PUA could be detected (Figure 1). This could be due to high cell lysis rates, causing PUA release with simultaneous disappearance of diatom cells due to cell death. Indeed, increased lysis rates have been estimated during *S. marinoi* blooms in this area [23]. In agreement with the concept of increased PUA production upon cell lysis, rapid bloom decay would result in the depletion of the particulate PUA and in increased concentration of dissolved PUA.

The observed concentrations of up to 0.1 nM PUA demonstrate for the first time that these compounds are released into the seawater and persist long enough to cause effects on the plankton community comparable to those observed in culture [7]. It needs to be considered that our measurements average over a quite large volume, while local concentrations in the immediate surroundings of the cells are probably higher due to a low diffusion process away from the producer [7]. The ecological consequences for organisms surrounding living or dying diatoms that release PUA would have to be estimated from local concentration determinations, but methods for microscale plankton investigations are currently not easily available.

## 3. Experimental Section

### 3.1. Field Sampling

A cruise was conducted in the northern Adriatic Sea in February 2008, on the RV Urania of the Italian National Research Council (CNR). Samples were collected using Niskin bottles mounted on a rosette sampler from the surface (0–1 m) between 15 and 20 February 2008 at the locations indicated in Figure 1. At selected locations (Figure 2) samples were also collected from deeper waters. Samples for phytoplankton counts were fixed with hexamethylenetetramine-neutralized formaldehyde to a final concentration of 4% and examined with an inverted microscope, equipped with phase contrast (model Zeiss Axiovert 35, Zeiss, Jena, Germany), at a final ×400 magnification. Sub-samples from 5 to 50 mL were allowed to settle for 12–48 hours and then examined by light microscopy. A variable transect number was observed until at least 200 (but often more than 500) cells of the most abundant species were counted for each sample.

Water samples were collected in polycarbonate bottles for the determination of particulate PUA of cells >1.2 μm 2 to 7 L of seawater, depending on cell density. Samples were filtered through GF/C filters (Whatman, Dassel, Germany). 1 L of the filtrate was filtered through 0.2 μm filters (Versapor-200, 47 mm, Pall life science, Port Washington, USA) in order to assess particulate PUA of cells between 0.2 and 1.2 μm in size. Both filters were treated with the freeze-and-thaw method described below, and stored at −20 °C until further analysis. For dissolved PUA analyses, 1 L of seawater was collected and filtered using the EASY cartridge enrichment method described below and stored at −20 °C until further analysis.

### 3.2. Apparatus

A GCT premier orthogonal reflectron time-of-flight (oTOF) mass spectrometer (MS) (Waters, Manchester, UK), coupled to an Agilent 6890N gas chromatograph (GC) equipped with a DB-5ms 30 m column (0.25 mm internal diameter, 0.25 μm film thickness, with 10 m Duraguard pre-column, Agilent, Waldbronn, Germany) was used for GC-EI-MS measurements. Helium 5.0 was the carrier gas with a constant flow of 1 mL min^−1^. The electron energy was 70 eV. Samples were injected with a 7683B autosampler (Agilent) equipped with a 10 μL tapered, fixed needle, PTFE-tipped plunger syringe (23–26s/42, Agilent). Calibration of the MS parameters (beam steering, focusing lenses, dynamic range extension (DRE)) was performed before analysis.

### 3.3. Reagents

High grade methanol (Chromasolv © Plus >99.9%, Sigma-Aldrich) and hexane (Suprasolv, Merck, via VWR) were used. *O*-(2,3,4,5,6-Pentafluorobenzyl)hydroxylamine hydrochloride (PFBHA, derivatization grade >99%, Sigma-Aldrich, or ABCR, Karlsruhe, Germany) was used for derivatization.

### 3.4. Data Analysis

The maps were generated with Ocean Data View (version 4.0.3, Schlitzer, R., http://odv.awi.de, 2010), color schemes in Figure 1 are based on extrapolations. Statistical analyses (Pearson’s correlation, T-test, One-way ANOVA followed by Holm-Sidak comparison, respectively) were conducted with SigmaPlot (version 11.0, Systat Softwares).

### 3.5. Determination of the Particulate PUA

Determination of the production of PUA by the cells was performed according to a modified protocol based on [24]. Cells were concentrated on a GF/C filter (Whatmann, Dassel, Germany) under vacuum (∼500 mBar), a protocol that was verified not to result the release of PUA from cells. The filter was then transferred to the inner surface of a 25 mL glass beaker. The cells were rinsed from the filter with 1 mL of a 25 mM PFBHA (Roth, Karlsruhe, Germany) solution in Tris-HCl 100 mM, pH 7.2. The cell suspension was then transferred to a 4 mL glass vial (Macherey-Nagel, Düren, Germany) and five microliters of internal standard (benzaldehyde, 1 mM in methanol, Sigma-Aldrich) was added. The vials were then closed with a screw cap fitted with butyl-PTFE septum (VWR, Dresden, Germany). For mechanical disruption the samples were frozen at −20 °C and thawed. The freezing/thawing was repeated 3 times and subsequently enzymatic reactions were allowed to proceed for 1 hour at room temperature. Samples were then kept overnight at 4 °C and stored at −20 °C. For extraction, 500 μL of methanol were added to each of the samples while still frozen. The samples were then allowed to thaw before addition of 1 mL of hexane. After vortexing for 1 min, 6 drops of sulfuric acid (Rotipuran©, >95%, Roth, Karlsruhe, Germany) were added with a glass Pasteur pipette (fitted with cotton wool to prevent contamination from the pipette ball). The samples were then vortexed for 1 min. Phase separation was achieved by centrifugation for 10 min at 1735 g at room temperature (in a Z383K centrifuge, Hermle, Wehingen, Germany). The hexane phase was transferred into 1.5 mL glass vials (Macherey-Nagel, Düren, Germany). After drying over anhydrous sodium sulphate, the hexane phase was transferred into new 1.5 mL glass vials. The solvent was removed under vacuum, and the samples were re-dissolved in 100 μL of hexane. After the transfer of the samples into 200 μL glass inserts (Macherey-Nagel, Düren, Germany), the vials were closed with caps fitted with PTFE-butyl-PTFE septa. The samples were then stored at −80 °C until analysis by GC-MS. The detection limit in this case cannot be determined, as it greatly depends on the cell density and volumes of sample that can be filtered. It is therefore different for each sample.

### 3.6. Determination of Dissolved PUA

Dissolved PUA concentrations were determined from ∼1 L using a modified protocol based on [11]. After exact volume determination the plankton samples were transferred into glass bottles and 5 microliters of internal standard (benzaldehyde, 1 mM in methanol) were added. EASY^®^ cartridges (Chromabond 3 mL, polar modified polystyrene-divinylbenzene copolymer, 200 mg, Macherey-Nagel, Düren, Germany) were loaded with 1 mL of a 25 mM PFBHA solution in Tris-HCl 100 mM pH 7.2 over a period of 5 min. A sand cartridge (empty Chromabond 3 mL cartridge filled with ∼3 mL of purified sea sand) was mounted inline before the EASY^®^ cartridge. The sample was passed through the two cartridges, which were connected via Teflon tubing, at a flow rate of *ca*. 1 L h^−1^. After washing with deionized water, the EASY^®^ cartridges were air-dried. The partially derivatized PUA were then eluted from the EASY^®^ cartridges into 4 mL glass vials using 4 mL of PFBHA in methanol (5 mM). The eluates were incubated for one hour at RT to ensure complete derivatisation and then stored at −20 °C. For extraction, the samples were transferred into 25 mL round-bottom glass flasks. Eight mL of hexane and 8 mL of distilled water were added before vortexing for 1 min. Sixty drops of sulfuric acid (Rotipuran©, >95%, Roth, Karlsruhe, Germany) were added with a glass Pasteur pipette and the samples were vortexed again for 1 min. The phases were allowed to separate, and the hexane phase was transferred into 10 mL round-bottom glass flasks. After drying over anhydrous sodium sulfate, the supernatant was transferred into 10 mL pointed-bottom flasks and evaporated under vacuum. The PUA-oximes were re-dissolved in 100 μL of hexane and transferred into glass inserts. The inserts were stored in 1.5 ml vials at −80 °C until GC-MS analysis.

### 3.7. Analysis and Quantification of PUA

Chromatograms were evaluated with the QuanLynx software (version 4.1, Waters, Manchester, UK). Identification of the PUA was based on the retention time compared with standards and the presence of the molecular ion (m/z 305 for heptadienal, 319 for octadienal, 315 for octatrienal, 347 for decadienal) as well as of major fragments ions (m/z 276 for the PUA, 271 for the internal standard, and 181 for all). The quantification was based on the ratio between the fragment m/z 276 of the derivatized PUA and the fragment m/z 271 of the derivatized internal standard (benzaldehyde), if the ion intensities were not saturating the detector. Otherwise, the ratios between the molecular ions of the PUA and benzaldehyde were used. A set of quantification standards was prepared before each analysis. To that end, 0.1, 0.2, 0.5, 1.0, 5.0, 10.0 or 20.0 nmol of heptadienal (>97%, Sigma-Aldrich), octadienal (96+%, Sigma-Aldrich) and decadienal (85%, Sigma-Aldrich) were added from 1 mM methanolic solutions into 1 mL of a 25 mM PFBHA solution in Tris-HCl 100 mM pH 7.2. After addition of 5 μL of internal standard (benzaldehyde, 1 mM in methanol), the samples were incubated for 1 hour at RT, before being extracted according to the above mentioned procedure. Samples were run in random order after the quantification standards. The GC temperature program for the separation was 60 °C (2 min) then increased with a rate of 8 °C min^−1^ to 240 °C and then with a rate of 15 °C min^−1^ to 280 °C (2 min).

## 4. Conclusions

We show for the first time that PUA are released into the seawater and persist there at sub nanomolar concentrations. PUA in the plankton were hitherto nearly exclusively associated with wound activated production processes by diatoms. As a consequence of our work novel roles of PUA as dissolved infochemicals can be considered in plankton interactions, supporting conclusions of culture studies. Further investigations on induced bloom termination events and on the influence of PUA on microbial communities could be motivated by our findings.

We also show that particulate octadienal appears to be a marker of *S. marinoi* in coastal seawater samples during blooms in the Northern Adriatic Sea. In contrast, additional cells in the size range between 0.2 and 1.2 μm have to be taken into account for particulate and dissolved heptadienal. This PUA appears to be more widespread than previously thought and additional organisms producing it must still be identified.

## Figures and Tables

**Table 1.** Plankton cell densities and PUA concentrations on the sampled stations. Species are shown of the biomass on at least one station (the following species were present only on one station, *mediterraneus* (V33), *Rhizosolenia imbricate* (V28) and *Rhizosolenia setigera* (V37)).

Station	V4	V7	V8	V9	V12	V13	V13	V15	V17	V21	V21	V21	V28	V28	V33	V35	V37
Depth (m)	0	0	0	0	0	0	13	0	0	0	6	15	0	17	0	0	0
**Species and cell densities (×10^3^ cell L^−1^)**																	

**Bacillariophyceae^1^**	2.7	1.4	1.1	4.3	6.5	814	159.1	2.2	2.2	7.5	5.4	4.3	4.6	9.3	7	2.5	4.9

*Cerataulina pelagica*				2.2	4.3	37.7	58.6			2.2	1.1		0.8	3.6			
*Chaetoceros spp.*															2.2		3.5
*Guinardia flaccida*				2.2							0.5						
*Pseudosolenia calcar avis*								1.1					0.3	0.7		0.4	
*Skeletonema marinoi*						776.3	71.2				0.5		1.6	2.2			
*Thalassiosira rotula*										2.2			1.3	0.7			

**Dinophyceae^1^**	2.2	135.1	35.6	112.1	114.3	237.2	184.2		0.5	29.1	5.9	0.5	7.5	11.5	1.6	1.1	1.6

*Gonyaulax polygramma*				8.6									0.3				
*Gymnodinium spp.*		11.5		4.3	15.1	16.2	16.7		0.5				3.2	0.7	1.6		1.6
*Prorocentrum minimum*	1.6	122.2	20.5	15.1	81.9	221	142.3			27	5.9	0.5	3	9.3			
Und. naked	0.5		9.7	10.8	6.5		8.4									0.7	

**Prymnesiophyceae^1^**	15.1	10.1	21.6	56.1	30.2	5.4	4.2	18.6	33.4	1.6	16.7	17.3	12.7	27.3	6.5	16.2	11.9

*Emiliania huxleyi*	15.1	10.1	21.6	38.8	30.2	5.4	4.2	18.6	33.4	1.6	16.7	17.3	12.7	27.3	6.5	12.2	11.3

**Cryptophyceae^1^**	11.3	46	18.3	97	28	248	12.6	5.7	9.2	39.9	18.3	5.4	13.5	18	19.4	4.3	3.2

**Nanoflagellates^1^**	23.7	140.9	139.1	394.6	472.2	690	493.9	30.2	53.9	194.1	124.5	201.6	108.1	92	51.2	75.1	52

**Total^1^**	55	333.5	215.6	664.1	651.2	1994.6	853.9	56.6	99.2	272.2	170.9	229.1	146.4	158.1	85.7	99.2	73.6

**Dissolved PUA (nM)**																	

Heptadienal	0.032	0.03	0.039	0.019	0.028	0.079	0.128	0.033	0.022	0.059	0.062	0.051	0.053	0.049	0.046	0.054	0.041
Octadienal	0.043	0.039	0.027	0.032	0.005	0.118	0.109	0.048	0	0.055	0.037	0.05	0	0	0	0	0

**Particulate > 1.2 μ (nmol per cells from 1 L, ±Standard deviation, n = 3)**																	

Heptadienal	0	0	0	0	0	18.1±7.1	7.9±1.1	0.04±0.01	0.05±0.02	3.61±0.4	0.29±0.091	0.22±0.14	0.41±0.08	0.13±0.02	0.02±0.02	0.03±0.02	0
Octadienal	0	0	0	0	0	10.5±2.7	4.5±0.9	0	0	1.62±0.07	0.074±0.033	0.05±0.06	0.1±0.08	0	0	0	0

**0.2 < Particulate < 1.2 μ (nmol per cells from 1 L)^2^**																	

Heptadienal	0	0	0.106	0	0	0	0.043	0.064	0.635	0.038	0.043	1.804	0.202	0.015	0	0.046	0.041

1 values are the total of all counted cells for the group, including the species not presented here.

2 no octadienal detected in these samples.
